# Clinical Remote Monitoring of Individuals With Spinal Cord Injury at Risk for Pressure Injury Recurrence Using mHealth: Protocol for a Pilot, Pragmatic, Hybrid Implementation Trial

**DOI:** 10.2196/51849

**Published:** 2024-04-10

**Authors:** Melissa M Morrow, Lynne C Hughes, Diane M Collins, Tamara L Vos-Draper

**Affiliations:** 1 Department of Physical Therapy & Rehabilitation Sciences University of Texas Medical Branch Galveston, TX United States; 2 College of Pharmacy Program in Occupational Therapy University of Minnesota Minneapolis, MN United States

**Keywords:** wheelchair user, seating and mobility, weight shift behavior, pressure injury, mobile phone

## Abstract

**Background:**

Pressure injuries are one of the most challenging secondary conditions for individuals with spinal cord injuries and related disorders (SCI/D) owing to inherent, lifelong risk factors that include a lack of sensory and motor function below the level of injury and reliance on a wheelchair for daily mobility, resulting in prolonged periods of sitting. Although many factors contribute to the development of pressure injuries, the pressure between the skin and a surface is always a factor and the development of injury is dependent on the magnitude and duration of the pressure. Clinically, broad recommendations for relieving pressure are used because we know very little about the unique day-to-day life patterns of the individual wheelchair user. Typically, it is after the occurrence of a pressure injury that the therapist will check equipment fit and the effectiveness of pressure offloading and ask about other surfaces they sit on in their home and community. This time-lapsed, largely self-reported data are fraught with recall bias and inaccuracies that the therapist incorporates into a plan of care.

**Objective:**

This study’s objective is to pilot-test the implementation and clinical effectiveness of a telehealth model of care combined with our mobile health (mHealth) Assisted Weight-Shift device for remote monitoring of factors related to maintaining skin health and wheelchair setup. Our overall hypothesis is that this study will result in an effective implementation plan, and the enhanced connected model of care using remote monitoring of pressure management will result in pilot-level, improved clinical outcomes for adults with spinal cord injury at high risk for pressure injury recurrence.

**Methods:**

For all aims, we will use a mixed methods design using an exploratory, sequential approach to include the strengths of both qualitative and quantitative data. For aims 1 and 2, we will iteratively collect qualitative data from therapists, patients with SCI/D, and other stakeholders. For aim 3, we will perform a hybrid effectiveness-implementation randomized controlled trial to pilot-test the intervention. The projected results include an iteratively developed and tested implementation plan that meets moderate to high levels of acceptability, feasibility, and appropriateness. Additionally, the pilot trial results are expected to show positive trends in relevant clinical outcomes related to reduced pressure injury incidence, recurrence, and improved healing when compared with the standard of care.

**Results:**

Currently, 6 participants have been recruited for our aim-1 qualitative study.

**Conclusions:**

This study will expand upon our previous study to move the Assisted Weight-Shift system into routine clinical care, which was a strong desire of adults with SCI/D for improved individualized care plans to prevent pressure injuries. The results of this study will guide the next steps in a full, hybrid effectiveness-implementation trial with the goal of improving care to prevent pressure injuries.

**International Registered Report Identifier (IRRID):**

DERR1-10.2196/51849

## Introduction

### Background

Pressure injuries are one of the most challenging secondary conditions for individuals with spinal cord injuries and related disorders (SCI/D) owing to inherent lifelong risk factors that include a lack of sensory and motor function below the level of injury and reliance on a wheelchair for daily mobility, resulting in prolonged periods of sitting. A 2022 report stated that the incidence of pressure injuries in adults with SCI/D was 25% to 38%, with incidence increasing with time after SCI/D onset [1,2]. Recurrence rates as high as 80% [3-5] have been reported. Pressure injuries are also the second most common reason for rehospitalization at a rate of 11% to 20%, and these numbers are also increasing with time since SCI/D onset [1]. Pressure injuries are the only secondary condition consistently correlated with mortality risk [1,6], and they cause a significant negative impact on the individual, health care system, and society owing to the costs to manage them once they are present.

### Interface Pressure Is a Major Risk Factor for Pressure Injuries

Although many factors contribute to the development of pressure injuries [7,8], the pressure between the skin and a surface is always a factor and the development of injury is dependent on the magnitude and duration of pressure [9,10]. An individual’s pressure versus time curve is based on their ability to physiologically tolerate mechanical loads over time [11]. The longer the pressure is applied without mitigation, the higher the risk for tissue damage. Some individuals can tolerate more load over longer periods of time than others; therefore, individualization is important for prevention.

### Individualized Solutions Are Necessary for Effective Prevention

Unique risk factors are present for individuals with SCI/D, including motor and sensory impairment; changes to muscle and bone tissues; and for up to 80% of those with SCI/D, the reliance on a wheelchair for mobility [1]. Individuals with more complete injuries have more impaired sensory function [12] and tend to fare worse in their ability to prevent pressure injury over time [13-15]. Movement is a key mechanism in offloading pressure, and there is high variability in daily movement patterns across the SCI/D population [16], which makes it difficult to standardize the recommendations for performing weight shifts. In addition, some individuals are physiologically better able to tolerate more or longer duration of pressure than others [17]. Each person has unique levels of risk awareness, daily routines, habits, and contexts within which they function, and these influence their ability to adhere to preventative behaviors [18]. Thus, for preventative behaviors to be added successfully to daily life, they need to be customizable and individualized to fit within each person’s unique scenarios.

### Standard of Care for Pressure Injury Prevention Is Individualized but Includes Limited Data About Daily Life

For the occupational or physical therapy seating specialists, care is provided in a client-centered manner such that education, treatments, and recommendations are individualized to the person as much as possible. This client-centered approach includes education about minimizing prolonged pressures under bony areas [3,11,19,20], with movement strategies to redistribute pressures [21-26] that are specific to the individual’s injury level, functional level, body habitus, and so on. However, although the wheelchair, seating surface, and mechanism of pressure offloading (leaning or tilt) are specialized to the person, the clinical recommendations for the frequency and duration of skin-protective movement patterns are broadly applied to all people and range from performing weight shifts every 15 minutes to every hour and holding them for up to a 2-minute duration [3,7,19]. Broad recommendations are used because we know very little about the unique day-to-day life patterns of the individual wheelchair user. If a patient returns to their therapist after the occurrence of a pressure injury, the therapist can check equipment fit and the effectiveness of pressure offloading, and they can ask about other surfaces they sit on in their home and community. This time-lapsed, largely self-reported data are fraught with recall bias and inaccuracies that the therapist does their best to incorporate into an effective care plan.

However, based on the rates of pressure injury incidence and recurrence, there is a gap in the care we can provide to individuals with SCI/D. We hypothesize that this gap is largely owing to the limited availability of data about the patient’s daily life. On the basis of our own preliminary data, we know that therapists want to know the following objective and contextual data about their patients: (1) frequency and duration of daily pressure offloading behaviors, (2) effectiveness of pressure offloading during their daily weight shifts, and (3) pressure distribution on the other sitting surfaces the patient interacts with (chair, car seat, commode, etc). Without the necessary client-centered data gathered in context, the therapist is significantly limited in developing an individualized care plan.

### The Assisted Weight-Shift System Monitors Factors of Individualized Pressure Management

**Figure 1:**
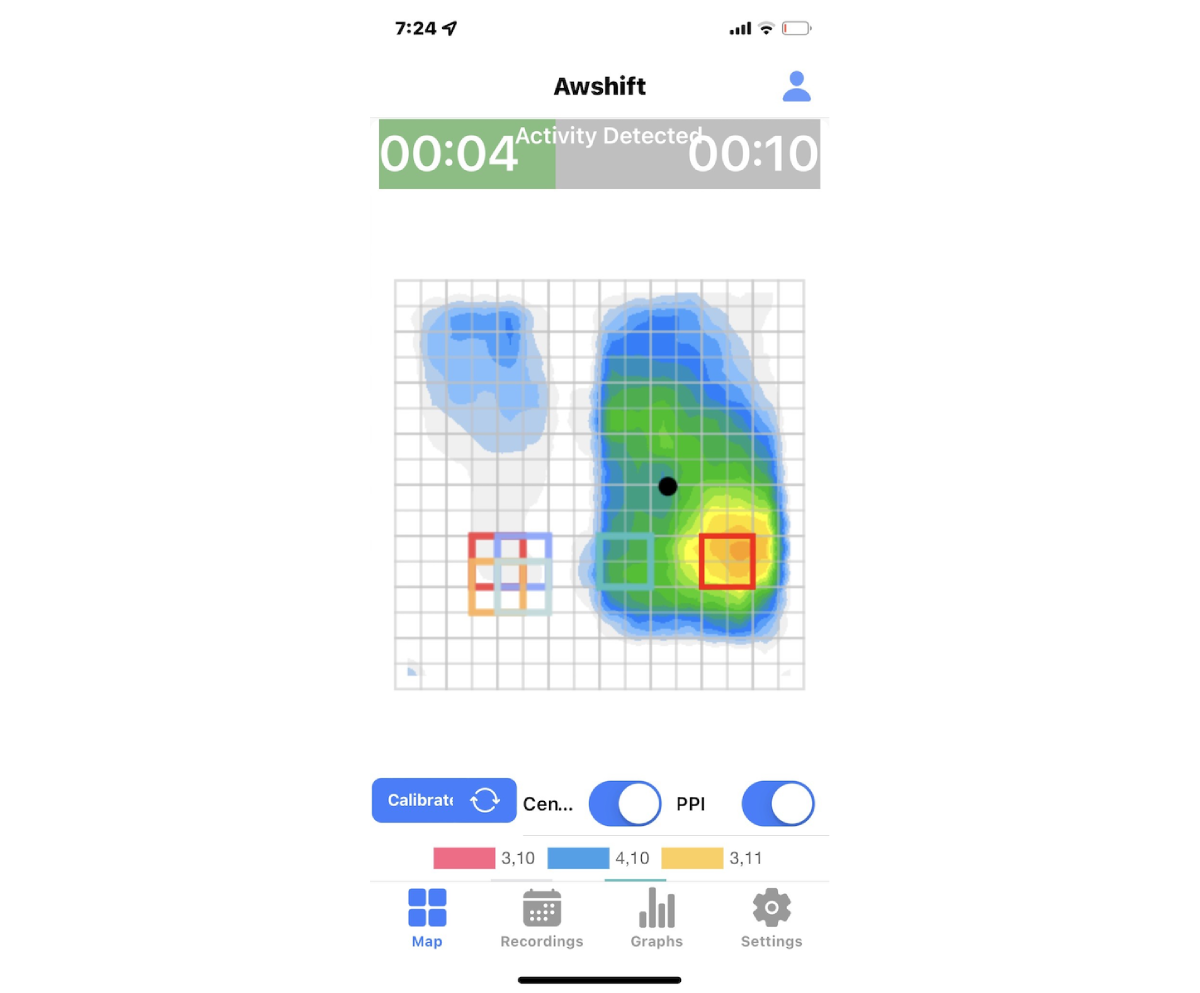
Assisted Weight-Shift (AW-Shift) real-time pressure map and countdown to next weight shift. PPI: Peak Pressure Index.

In our Department of Defense (DoD) Spinal Cord Injury Research Program–funded project (W81XWH-15-1-0484), our group, in collaboration with veterans with SCI/D, developed the Assisted Weight-Shift (AW-Shift) system (formerly called the comprehensive mobile assessment of pressure system) to capture objective data regarding pressure management during daily life to be used by the patient or consumer and clinical partners to better prevent pressure injuries [27]. In a current National Institute of Health (NIH)–funded project (R01 AG056255), we have improved the hardware and software design of the AW-Shift system and are performing a clinical trial of the system in a consumer-use model. The AW-Shift system provides real-time pressure mapping visualization (Figure 1), tracks weight shift behaviors, and provides weight shift reminders and high-pressure alerts. The data provided by the AW-Shift system offer unique, client-centered information to drive individualized goals and intervention plans. With this level of information about daily life, each person’s unique daily occupations, routines, and rituals that influence their pressure management profile can be directly integrated into individualized care. Through DoD and NIH funding, we have demonstrated the efficacy of the system in terms of improving pressure injury prevention awareness, self-efficacy, and pressure management behavior in a consumer-use model. The proposed project advances our past studies through implementation of the AW-Shift system and evaluation of its impact on the clinical care of adults with SCI/D.

### Structured Implementation Studies Are Necessary for Clinical Practice Improvement

Implementation research is the study of methods to optimize the systematic uptake of evidence that has been shown to be efficacious (has the intended effect) and effective (has the intended effect in the intended setting). As noted, emerging technologies such as AW-Shift may provide safe, efficient, and effective pressure injury prevention, but additional studies are needed to determine what approaches are optimal for integrating this technology and individualized data into clinical practice. The implementation of the AW-Shift system into routine clinical practice is far more complex than simply providing proof that the system works in a research setting.

Implementation into practice requires the commitment and active involvement of practitioners and patients, collaboration with relevant stakeholders who influence care delivery, and modifications to the intervention that meet the needs of providers and patients in dynamic practice settings. We have designed this project to assess the current clinical practices, iteratively modify the intervention to meet the clinical practice needs, and identify what factors may influence its uptake.

### Overall Objective and Specific Aims

#### Overview

Our long-term objective is to facilitate the effective prevention of seating-related pressure injuries throughout the lifetime of individuals with SCI/D with the use of our evidence-based, real-time pressure management solution, the AW-Shift system (Figure 1). Our short-term objective for this study is to pilot-test the implementation and effectiveness of a telehealth model of care combined with our mobile health (mHealth) AW-Shift device for remote monitoring of factors related to maintaining skin health and wheelchair setup. Our overall hypothesis is that our development plan will result in an effective implementation plan, and the enhanced connected model of care using remote monitoring of pressure management will result in pilot-level improved clinical outcomes (wound recurrence, early identification of wounds, and wound healing) for adults with spinal cord injury (SCI) at high risk for pressure injury recurrence. We will pursue our objective through the following specific aims.

#### Aim 1: Clinical System Development (Develop mHealth Report and Integrate Within the Medical Electronic Environment)

The current remote monitoring interface was built for research use. Through a user-centered, mixed methods design, we will engage with patients and clinicians through focus groups, interviews, and surveys to determine the optimized design for the capture and reporting of data to the patient and the clinician through a connected care model. After the capture and reporting method is developed, the data flow will be integrated within electronic medical record infrastructure.

#### Aim 2: Rapid Cycle Quality Improvement (Integrate System Into Connected Care and Telemedicine Infrastructure)

Using best practices for rapid cycle quality improvement, we will integrate our connected care solution into a current telehealth infrastructure. We will test the ability of our connected care solution to perform patient visits and short-term remote monitoring of pressure management focused on the system integration aspects to cycle through improvements in a 3-month time frame to prepare for aim 3.

#### Aim 3: Pragmatic, Hybrid Implementation Trial (Implement the Connected Care Solution via a Pilot, Pragmatic Trial Comparing Clinical Outcomes Between the Connected Care Model and Standard Practice)

**Figure 2:**
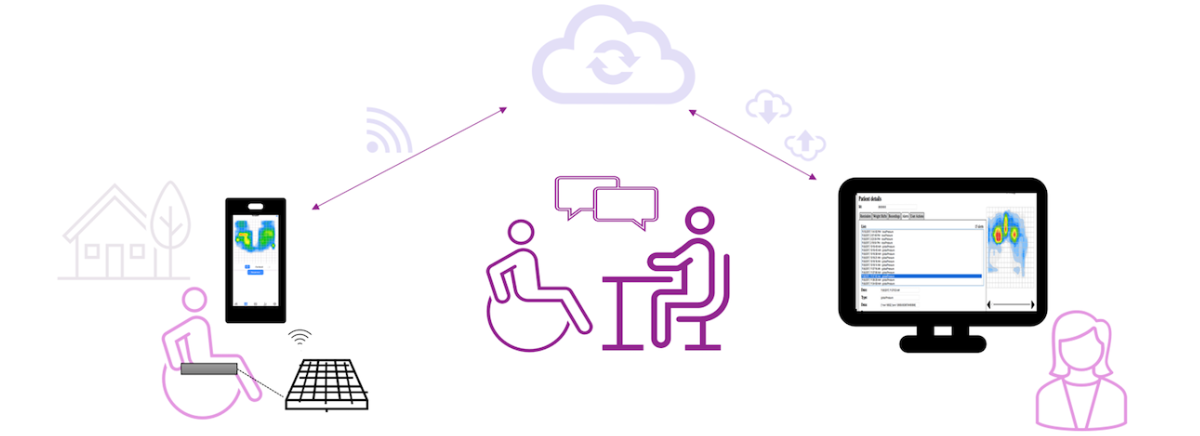
Through a connected care model and the Assisted Weight-Shift mobile health system, the provider guides the patient to change high-risk pressures (phone image on the left) into safe pressures (phone image on the right).

Patients identified as having high risk for pressure injury recurrence will be randomized to either the connected care model (Figure 2) or the standard model of care for a 1-year follow-up time frame. Our implementation outcomes include important stakeholders’ assessment of feasibility, acceptability, appropriateness, costs, and improved monitoring. Our pilot effectiveness outcomes are rate of wound healing for prevalent cases, incidence and recurrence of pressure injuries, early identification of wounds, and hospital admissions or readmissions.

## Methods

### Study Design

For all aims, we will use a mixed methods design using an exploratory, sequential approach to include the strengths of both qualitative and quantitative data. For aims 1 and 2 of the study, in an iterative manner, we will collect qualitative data from therapists, patients, and other stakeholders. For aim 3, using both qualitative and quantitative data, we will perform a hybrid effectiveness-implementation randomized controlled trial to pilot-test the intervention. For implementation outcomes, we will assess the feasibility, acceptability, and appropriateness for the in-home use by the patients and the feasibility, acceptability, and appropriateness of the previsit information from clinical stakeholders. For pilot effectiveness, we will assess the clinical outcome measures of wound recurrence, early identification of wounds, and wound healing.

### Ethical Considerations

All research activities for aims 1 to 3 will be performed under institutional review board (IRB) oversight and with IRB approval. At the time of the writing of this manuscript, aim 1 was approved by the University of Texas Medical Branch IRB (22-0280) as an exempt study that waives the requirement for informed consent. The study is no more than minimal risk and fits the second exemption category (interviews), including recording data in such a manner that the identity of the human participants cannot be readily ascertained through identifiers linked to the participants. IRB approval for aims 2 and 3 will be sought under the minimal risk category. We expect that owing to the iterative nature of aims 2 and 3, modifications will be required throughout the study to accommodate updates to the protocol and procedures, motivated by study staff and participant experiences and feedback. Participants for aim 1 are compensated for their participation with a gift card worth US $50. Participants for aims 1 and 2 will be compensated for their time with payment commiserate with the time required to complete the study.

### Rationale for Hybrid Effectiveness-Implementation Study Design

Hybrid designs were developed to improve efficiency in moving promising interventions along the efficacy-effectiveness-implementation continuum, as implementation research focuses on the adoption of evidence-based interventions by systems of care. Integrating implementation planning into the study design helps to promote the full adoption of an intervention into the practice in which it was tested. We propose to use a hybrid-1 design [28], wherein the study is designed to test a pilot clinical intervention while gathering information about its delivery during the effectiveness trial and about its potential implementation in “the real world” of clinical practice. We will simultaneously plan for implementing AW-Shift into practice while testing its effectiveness in changing clinical outcomes. Our design includes formative evaluations of clinical staff during the study period, which will provide a rigorous assessment of what potential internal and external influences are critical for a successful implementation and add qualitative data to enrich the data analysis and evaluation.

### Rationale for Using Consolidated Framework for Implementation Research Implementation Model to Guide Implementation

We propose to use Consolidated Framework for Implementation Research (CFIR) to inform which implementation targets will be critical for successful implementation. CFIR is a meta-theoretical framework that is based on a synthesis of 19 implementation frameworks and models with a goal to “foster knowledge-building into why implementations succeed or fail.” It includes 5 domains: outer setting, inner setting, intervention characteristics, individual characteristics, and process. Each domain includes common constructs, 39 in total, associated with successful implementation. The framework does not specify hypotheses or causal pathways, allowing flexibility in evaluating constructs that are unique to a study. It has been described as a “menu of constructs” that enables systematic and comprehensive exploration and identification of potential factors that can influence implementation [29]. In general, the framework helps researchers and interventionists to assess the structural capacity for an intervention, culture for change, perceptions from stakeholders about the need for change, quality of the proposed solution, and way in which the intervention should be planned and executed. Regarding the qualitative work for aim 1, we will explore the potential constructs in each domain (eg, design quality and packaging, patient needs and resources, compatibility, relative priority, leadership engagement, and self-efficacy) from the CFIR model that will inform the efforts for the rapid cycle assessment in aim 2 and pilot trial in aim 3.

### Specific Aim-1 Methods: Clinical System Development

The goal of aim 1 is to define the scope of the implementation of the AW-Shift system into a connected care model and operationalize that scope into a testable plan for aim 2. Each step of the plan will be guided by the end users and other key stakeholders through qualitative and survey feedback.

#### Patient and Clinician Needs Assessment

Overall, 2 focus groups, 1 for adults with SCI/D and 1 for clinicians from the Seating Clinic and Wound Care, will be conducted to determine the needs and priorities of each group for improving the assessment of seating and mobility needs. A focus group guide, drawn from CFIR’s Interview Guide Tool [30], will be used for each group to better understand how expanded seating pressure information captured during the daily life of patients could better inform seating and mobility therapy visits. The discussion will include brainstorming of types of information that would be of value and identifying barriers to and facilitators of attaining the information. There will be an emphasis on understanding the concerns about the complexities of a new process as it relates to the outer setting, inner setting, and individual characteristic constructs of the CFIR model [31]. The focus groups will be conducted through a Health Insurance Portability and Accountability Act–compliant web-based meeting format (Zoom; Zoom Video Communications). The discussion will be recorded for analysis.

#### Qualitative Outcome Measures and Data Analysis

The qualitative data from the focus groups will be transcribed by a transcriptionist. Investigators will import transcripts and field notes into computer-assisted coding software such as NVivo (Lumivero) to structure the transcripts and enable them to be “openly coded” by the researchers. The CFIR constructs will guide the development of the code book, as these constructs are key to effective implementation. Qualitative analysis of the interview transcripts will combine deductive and inductive approaches using an initial coding template structured around the interview guide topics and then refined through full data set coding [32]. The coding template refinement process will include an element of reflexivity owing to clinical expertise of coders, thus offering an efficient process to generate high-value design requirements layered with contextual rationale. Through memo-taking and iterative meetings to discuss data, the team will reach consensus about the code book and the assignment of text segments to particular codes. Investigators will independently review the transcripts and codes to provide a measure of internal validation. A coalescence of major themes from the coders will be used for the interpretation of findings and the subsequent theory development. The survey data will be used to define the areas of strengths and weaknesses of the intervention and implementation plan.

#### Sample Size

For focus groups, we will follow the recommended practice by having no more than 10 participants per group [33]. For the patient focus group (n=10), we aim to purposively sample patients with various levels of SCI/D, wheelchair type (power and manual), history of pressure injuries, and age to seek a wide breadth of user experiences. For the therapist focus group (n=10), we aim to purposely sample therapists with a range of years of experience, to seek a breadth of perspectives about providing seating and mobility care to patients with SCI/D.

#### Draft Plan and Follow-up Focus Group

**Figure 3:**
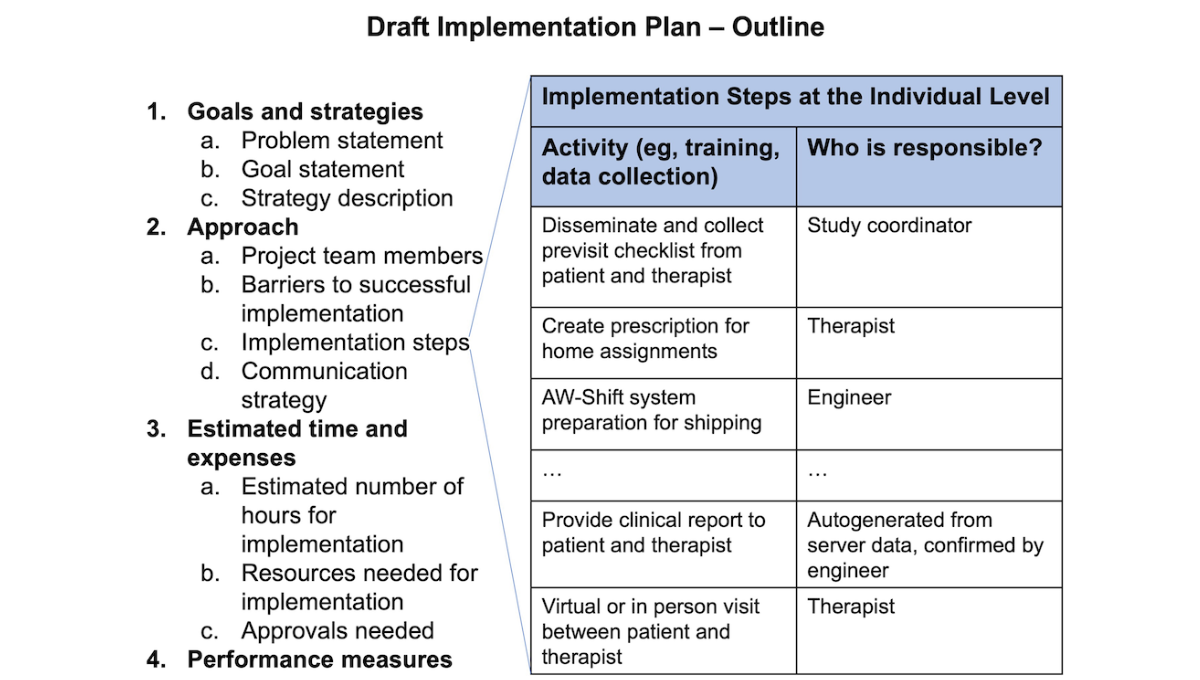
Implementation outline with example implementation steps. AW-Shift: Assisted Weight-Shift.

Needs assessment will be used to refine the draft implementation plan (Figure 3). Following the refinement of the draft plan, a follow-up focus group with clinicians and patients will be conducted, wherein the draft plan will be presented along with a description of the AW-Shift system. Through this focus group, we seek to understand the specific types of data and activities to measure during daily life that can inform the clinical assessment. In addition, the participants may be queried about specific aspects of the plan using survey responses that will likely use a Likert scale for assessing the level of satisfaction or acceptability with aspects of the plan. We will learn from these focus groups and survey responses regarding what needs to be adapted in the draft plan and what data will be used in the mHealth report. We will operationalize the implementation plan based on the participant feedback and use the CFIR constructs to increase the usability and acceptance of the AW-Shift system by participants or patients with SCI/D.

#### mHealth Report

**Figure 4:**
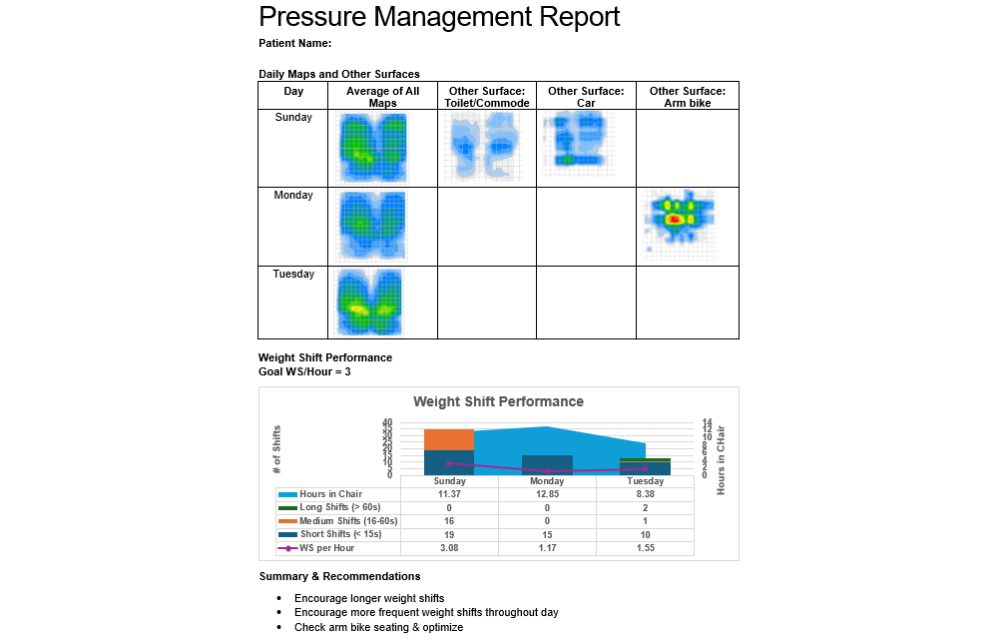
Example of a partial mobile health report. AW-Shift: Assisted Weight-Shift.

The mHealth report will provide summary data and visualizations that provide an informative snapshot of the home testing period (a partial example is shown in Figure 4).

#### Integration of the mHealth Report Into the Clinical Electronic Environment

On the basis of the focus group findings, the team will perform the following tasks in collaboration with necessary stakeholders. We will adapt the current, remote data capture process and infrastructure to meet the patient and therapist priorities and requirements for use during the clinical visit. A likely solution for communication of the data will involve the mHealth report being autogenerated on the server and provided back to the patient through the mobile app before the telehealth visit. The patient, being the ultimate owner of their data, will then send their report to their therapist through a secure patient website or mobile app that allows direct communication to the care team and patient access to their web-based medical record. With this data flow plan, the report would be available to the therapist for easy viewing during their visit. In addition, the patient will have full awareness of the data that the clinician is viewing.

#### Iterative Process for User-Designed Implementation of the AW-Shift System Into Clinical Care

Through a series of iterative focus groups and further refinement steps, the team, along with the users of the system (patients and therapists), will co-design the full implementation plan that will be ready for testing in aim 2. We estimate this step will take 2 to 3 iterations of focus groups and refinement steps.

### Specific Aim-2 Methods: Rapid Cycle Quality Improvement

#### Overview

Rapid quality improvement assessment of the implementation plan will be completed in aim 2 by testing the plan with an estimated 10 patients with SCI/D. For the first 3 to 5 patient participants, each participant will be run 1 at a time through the implementation plan. Rapid adjustments will be made as each participant goes through the steps of the plan. For the final 6 to 8 participants, participants will be scheduled in pairs such that at least 2 participants will be run in parallel to test the ability of the implementation plan to adjust to concurrent use of the system in the participants’ home and community. After each participant set, we will make rapid adjustments to optimize the implementation plan. Owing to participant heterogeneity, we will be mindful about how plans will need to be optimized differently depending on patient characteristics such as injury level and whether a caregiver or personal aid is required by the patient to use the system.

#### Protocol for Connected Care Model

Although the specific details of this protocol may change, we propose an outline of the procedures that will occur after participant consent and enrollment.

#### Individualized AW-Shift Prescription

A seating clinic therapist will review the clinical notes of the participant for their seating and mobility history. We presume from our preliminary data from therapists and the knowledge of the clinical team members in this project that a simple initial checklist will be effective in determining the possible “home assignments” that therapists could give to patients to gather the pertinent information specific to the patient, including recording pressure across different surfaces, using weight shift reminders, and recording weight shifts performed during specific tasks. In addition, the patient will be asked to complete a checklist to identify their seating and mobility concerns including, for example, new areas of concern about their skin, seat cushion, weight shifting habits, pressure on other surfaces, sitting posture, or weight relief quantity or quality. On the basis of the checklist information, an individualized prescription will be developed for the participant to fulfill (with extensive team support) during the 2-week daily life testing period.

#### System Setup and Prescription Delivery

The participant will be shipped an AW-Shift system and scheduled for a virtual visit to set up the system by the study coordinator. The system will include the hardware components, a user manual, and a study-provided smartphone or tablet if the participant does not have their own device. If the participant is using their own phone, they will be instructed to download the AW-Shift mobile app from the Apple App Store or Google Play Store. During the system setup visit, the participant will be provided with training regarding how to use the AW-Shift system with the study coordinator and engineering and therapy experts on the study team. The team will review the individualized prescription with the participant, and knowledge about system use will be tested to determine sufficient mastery of using the system.

#### Home and Community Use of the AW-Shift System

Participants will use the system for an estimated 2 weeks before their clinical visit. Participants will have easy access via phone; secure, web-based format; email; and SMS text message for technical and therapy support. A potential prescription could include (but not be limited to) mapping the variety of surfaces the participant sits on in the home and community (the system can be used on any surface), completion of daily weight shift protocols to assess participant positioning on their cushion, measurement of how the participant performs weight shifts with and without reminders provided by AW-Shift, and measurement of the effectiveness of the weight shifts to offload pressure.

#### Summary Report

Before the telehealth clinic visit, the prepared mHealth report will be delivered via the AW-Shift mobile app to the participant. The participant will be instructed about how to upload the report 1 day before their telehealth clinical visit. The therapist will view the mHealth report via the participant’s medical records during the clinical visit.

#### Telehealth Clinical Visit

During the visit, the therapist will use the standard clinical notes and the data provided on the mHealth report to guide the patient participant through the typical steps of a therapy visit focused on the seating and mobility needs related to pressure injury prevention. The telehealth visit will be completed using the standard Zoom interface. The therapist will use all the information they have available to determine the next steps in the patient’s care plan. The clinic visits will be recorded, so that the study team can provide additional observations for use in process improvement. The therapist will ask for confirmation of a skin check performed on the day of the visit with assistance from a caregiver, if necessary.

#### Assessment

Following the telehealth visit, both the patient participants and the therapists will complete a series of surveys to assess usability (System Usability Survey [34]), acceptability (Unified Theory of Acceptance of Use of Technology [35,36]), feasibility (Feasibility of Intervention Measure [37]), and appropriateness [37] of the procedures, and each of them will participate in a short interview to gather qualitative feedback to improve the implementation plan.

#### Rapid Assessment Procedure

Rapid assessment procedures are a pragmatic option for producing timely, contextually rich evaluative information about complex interventions implemented into dynamic clinical settings [38]. We will use rapid assessment procedures after each participant in the first 3 to 5 participants or after each pair of the final 6 to 8 participants to improve the plan for the next set of participants.

#### Stakeholder Input About Case Summaries

The findings from aim 2 will be written as a case summary that will be presented to the patient and therapist focus groups from aim 1 to gather additional stakeholder input about the optimization process. Any points regarding necessary improvement or inconsistencies will be discussed by the research team until consensus is achieved for the final plan for aim 3.

#### Outcome Measures and Data Analysis

The qualitative data from focus groups and interviews will be managed and analyzed as described for aim 1. The quantitative data from the survey scores will be used to define the areas of strengths and weaknesses of the intervention and implementation plan for further refinement.

#### Sample Size

For the rapid assessment testing for aim 2, we provide a robust approach by cycling the process through single and paired visits. Similar to the focus groups, we will sample to attain a breadth of user experiences. A total of 10 participants for each round is an effective sample size to be able to narrow down and optimize the process [33].

#### Data Management

All study data will have unique identifiers removed before the final analysis. Study data will be imported from the various clinical data sets into a central REDCap (Research Electronic Data Capture; Vanderbilt University) database that can be accessed by the study team. The study coordinator managing the database will keep the electronic key secure in accordance with Health Insurance Portability and Accountability Act and patient privacy regulations.

#### Potential Limitations and Alternative Strategies

##### Failure of the System in the Field

We will monitor system use and will be available for troubleshooting. We currently have 25 full systems and will be able to accommodate equipment swapping by mail.

##### Recruitment

We have experienced very little difficulty in reaching our goals for participation in past studies with similar methodology. We will continue to collaborate closely with clinical staff and consumer groups to ensure successful recruitment.

##### Skin Safety

We will continue to track skin safety during the study and will discontinue use if we notice any signs of skin issues.

##### Sensing Technology

The current resistive sensors used in the selected pressure map have inherent limitations including a tendency to creep under prolonged loaded states, but we mitigate this through specific calibration protocols. The mat was selected because it stretches and is durable for whole day use.

### Specific Aim-3 Methods: Pilot

#### Pragmatic Hybrid Implementation Trial

The proposed pilot randomized controlled trial will test whether our novel AW-Shift system intervention adds value to the clinical care of adults with SCI/D to inform our next steps in translation.

#### Study Design

This single-site randomized controlled trial will use a modified intention-to-treat analysis. Randomization will be a stratified, block randomization with stratification based on wheelchair type (manual vs power) and current wound severity (no wound or stage 1 vs stage 2 or stage 3). The intervention group (26/52, 50%) will receive the connected care model that used AW-Shift in combination with telehealth. The control group (26/52, 50%) will receive the standard of care that uses telehealth when appropriate. The statistician will be masked to the group allocation information for the outcome measure analysis.

#### Randomization

We will stratify based on wheelchair type (2 groups: power and manual), and current wound severity (2 groups: no wound or stage 1 and stage 2 or stage 3). We will block randomize in groups of 4. This randomization scheme will ensure distribution of important confounders between arms of the study. Furthermore, the blocking will ensure equal distribution of group assignment during the study.

#### Study Population

Adults with SCI/D with high risk of recurring pressure injury who answer “yes” to one of the following questions are eligible: (1) I get pressure ulcers about every couple of years; (2) I get at least 1 pressure ulcer a year; and (3) I always seem to have sores, often requiring surgery or hospitalization [39]. Our rationale for choosing individuals at high risk for recurring injury is that once a person has one pressure injury, their chances are significantly high to have another; hence, it has been recommended that interventions focus on this group of individuals [39].

#### Initiating and Conducting the Pilot Clinical Trial

The principal investigators (PIs), coinvestigators, and study staff will manage the process of study initiation with a modified standard checklist that covers essential items. Pilot clinical trial initiation meeting will occur early in mid–year 1, wherein all the steps will be reviewed, and the specific timeline will be determined. Standard operating procedures and field guidebooks will be developed to follow throughout the trial period. Weekly phone calls or web-based meetings among all study members will be used for study management. Any urgent communication regarding the pilot clinical trial will be managed with a phone call to the PI made within 18 hours of the event and reported to the IRB.

#### Study Procedures

For initiation and administration of the intervention, the following procedures will be followed:

1. Project initiation: The project will be initiated following a study initiation checklist by the PI and local site investigators.
2. Staff training for the intervention: Staff will be instructed about how to use the AW-Shift system, view and use the mHealth report, and administer data collection elements as is appropriate for each study role. Data collection procedures will be standardized and tested for consistency across study team members.
3. Participant recruitment: Recruitment and enrollment during year 2 will occur primarily through clinical staff in collaboration with the study coordinator.
4. Intervention study period: The intervention will begin with 2 weeks of AW-Shift use at home by the patient with SCI/D, followed by a web-based clinical visit with a seating clinic therapist. Before mailing AW-Shift to participants, individualized tasks will be assigned by the seating specialist. The tasks are meant to inform the seating assessment process during the clinical visit. Participants and seating specialists will complete surveys after the seating clinic visit and participate in a guided interview.

#### Intervention Protocol

The planned intervention protocol is similar to that described for aim 2, for the rapid quality assessment as described in the *Protocol for Connected Care Model* section. We expect that the protocol will be improved through the iterative process we are using to optimize the implementation of our intervention. Full demographic variables relevant to SCI/D will be collected during the consent visit. The same surveys and structured interview will be completed after each visit by the intervention group participants and the therapists including assessment of usability (System Usability Survey [34]), acceptability (Unified Theory of Acceptance of Use of Technology [35,36]), feasibility (Feasibility of Intervention Measure [37]), and appropriateness [37] of the procedures.

#### Control Protocol

If participants are randomized into the control group, the standard of care protocol will be followed as used in the seating clinic. Demographic and additional variables will be collected from participants similar to those in the intervention group, and their clinical visit will be recorded for assessment of the visit.

#### General Statistical Methods

All tests are 2 sided, with a 0.05 type-1 error rate. Analysis will be conducted using SAS (SAS Institute) and R software (GNU Project). Mean and SD or median and IQR will be reported for all demographic, predictor, and outcome variables, as appropriate. For all outcome measures, the effect of relevant biological variables including sex, age, level of sensation, and pressure injury history will be assessed as covariates in the analysis. In addition, wheelchair type will be included as a covariate. Variables will be transformed as necessary to meet the appropriate distributional assumptions for modeling (eg, normality). For missing data, mixed models can be used to handle missing data naturally, so that all available data points may be analyzed, irrespective of whether the participant had some outcome data missing. However, analyses of data with multiple imputation for missing data may be attempted if the data are likely to be missing at random.

#### Primary Pilot Clinical Trial Effectiveness Outcomes

The primary effectiveness of the intervention will be captured via the collection of the following clinical outcomes: wound recurrence, early identification of wounds, wound healing, and hospital admission or readmission. The data for these metrics will be abstracted during the full year (365 days) after the study-related clinical visit for both the control and intervention group participants. Then, 2 independent therapist team members will abstract the medical records of each participant to search for the occurrence of an incident wound, stage at which wounds are identified, time frame of wound healing, and occurrence of a hospital admission or readmission directly related to a seating area pressure wound. Comparisons between groups for the primary outcome measures will be made using a 2-tailed *t* test. Additional comparisons will be made with regression and mixed effect models to explore the role of covariates on the study findings.

#### Secondary Pilot Clinical Trial Effectiveness Outcomes

**Figure 5:**
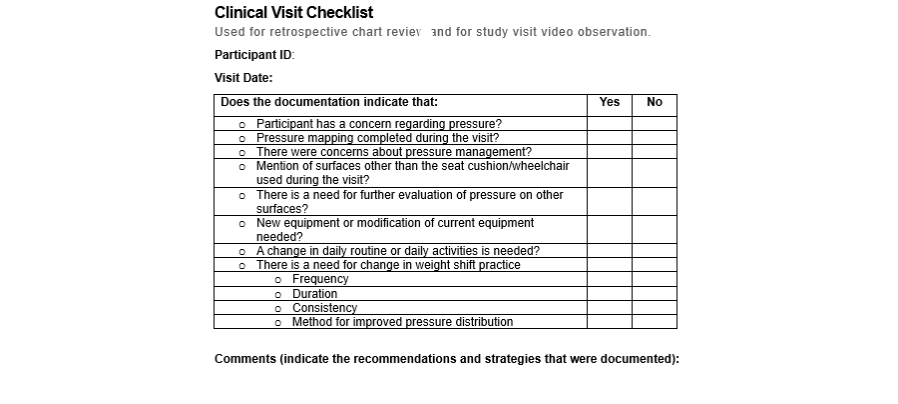
Clinical visit review checklist.

As a proxy outcome measure for clinical effectiveness, each clinical visit for the control and intervention groups will be assessed using the review of visit checklist shown in Figure 5 by the therapists leading the clinical visit. Then, 2 independent raters, who are seating specialists, will assess the checklist for each participant and will be blinded to the identities of the patients and therapist. The reviewers will rate the visit information on a 5-point Likert scale (eg, “Rate the effect of AW-Shift data on clinical recommendations: (1) significantly diminished, (2) diminished, (3) neither improved nor diminished, (4) improved, or (5) significantly improved”). The reviewer ratings will be analyzed with a 2-sample Mann-Whitney *U* test to determine if the ratings are different between groups to test our hypothesis that the AW-Shift data will significantly improve clinical recommendations. Effectiveness will also be assessed qualitatively in the interviews.

#### Implementation Outcome Measures

Implementation success will be assessed using feasibility, acceptability, and appropriateness surveys regarding the in-home system use of participants and therapists. This information will guide the broad implementation of the intervention.

#### Additional Study Variables, Controls, and End Points

Demographic variables relevant to SCI/D will be collected and used in the analysis as appropriate. Additional metrics include self-efficacy in managing pressure, assessed using the Skin Care Belief Scales [40]; consistency of weight shift performance, as measured by the AW-Shift system during home use; and the SCI–Quality of Life scale.

#### Feasibility Study Variables

For future clinical trial planning, the study variables of screening rate, recruitment rate, enrollment rate, and retention rate will be measured. In addition, the treatment adherence rate and treatment fidelity rates will be measured, and the assessment process will be evaluated. The assessment process includes documenting the planned assessments and data collection instruments that are completed in addition to the duration of the visits.

#### Sample Size

The sample size for this pilot study (N=52; n=26, 50% per group) is based on guidance that for a trial with 90% power and 2-sided 5% significance, pilot trial sample size of 25 participants per treatment arm is sufficient for an expected small, standardized effect size (0.2) [39]. We do not have the needed data for our primary outcomes to directly determine the power of our planned study design; however, this pilot study will be used to power the full clinical trial. Furthermore, we have used approaches to optimize our statistical power including blocking within the design itself that adjusts for covariates during randomization to reduce imbalance and triangulating the findings of quantitative analyses with qualitative data in mixed methods designs [41].

#### Potential Limitations and Alternative Strategies

In addition to the potential limitation described for aim 2, aim 3 has the added potential of loss to follow-up. As the long-term follow-up data are passively extracted from the medical record, we acknowledged that patients may seek care outside the original health system they were recruited from. In addition, for participants who have data that we cannot access, we will seek the approval of the participant to access their medical records from other medical systems.

### Human Participants

#### Study Population

Focus groups and structured interviews will be conducted for all aims. Qualitative methods will include adult patients with SCI/D and seating and mobility therapy specialists. Adults with a SCI/D will be the participants for aims 2 and 3. As needed, we will capture additional input from other stakeholders involved in the proposed implementation process to ensure that we are gathering views from a broad range of affected individuals as per the CFIR framework. These individuals may include therapy managers, IT staff, data security staff, scheduling staff, and industry representatives.

#### Recruitment

Following IRB guidelines, manual and power wheelchair users with traumatic or atraumatic SCI/Ds will be enrolled for the study. The inclusion and exclusion criteria were chosen to include wheelchair users with SCI/D who are at risk for pressure injury development. Recruitment will be purposeful in achieving the desired equal distribution of power and manual wheelchair users and individuals with paraplegia and tetraplegia. For study randomization in aim 3, we will use a stratified, blocked randomization method to ensure even distribution of important predictor and confounding variables in the intervention and control groups. Announcements for participation in this study will be made by personnel directly to patients through mailed letters, phone calls, and word of mouth. Upon determining a person’s eligibility, an appointment (via phone or in person) will be made with the study coordinator to explain the research project, confirm eligibility, and collect the necessary demographic data.

#### Inclusion and Exclusion Criteria

We will enroll adult men and women, aged between 18 and 80 years, with SCI/D at C4 or below, who have a lack or absence of sensation, are primary wheelchair users, and are at high risk for pressure injury. For aims 2 and 3, participants must have had a seating clinic visit or be scheduled for a seating clinic visit. Participants must demonstrate the ability to independently access the AW-Shift system app on a mobile phone or tablet and be able to participate in web-based visits or meetings with the research team. Participants will be excluded if they are unable to consent, have an active stage-4 wound at the seating surface site, do not have adequate resources or support for use of the AW-Shift technology in their home environment, or are currently receiving palliative care. We will enroll current occupational or physical therapy staff for aim-1 qualitative and survey feedback.

#### Potential Risks

There is the potential for skin irritation from sitting on a pressure mat for long periods. Participants may sit on the pressure mat throughout the day or may simply use it for short periods while performing their prescribed activities. Participants will be instructed to do a skin check every day and log their findings.

Participants are instructed to call the study coordinator if a new area of skin breakdown or wound occurs while they are using the pressure mat or if a current wound worsens. The participant will be asked to remove the mat until the skin breakdown or wound resolves. If the area does not resolve and worsens further, the participant will again contact the study coordinator and will then be referred to their typical clinical care provider in the case of a potential, new-onset incidence of pressure ulcers of stage 3 or 4. Initializing and deploying the AW-Shift system will require performing transfers and weight shifts. Transfers always have some risk of injury or fall, and the participant will be advised to transfer in their usual manner. Weight shifts will include typical functional movements, such as leaning forward and sideways, but participants will be instructed to only perform weight shifts with which they are comfortable. Risks from transferring on their own while the sensors are on the wheelchair are no different than transferring without the sensors present.

### Focus Group Guide

Questions were created for the clinicians’ structured interviews and focus groups. A sample of the question list is shown in Textbox 1.

**Sample of question list.**

- What are some of the challenges you come up against when teaching a patient with spinal cord injuries and related disorders (SCI/D) about pressure relief?
- On the basis of feedback from our patients of how very useful real-time information is to their pressure relieving behavior. What do you think about the having the real-time image available all the time to the patient?
  Do you think having that image available will assist you in monitoring and teaching seating behavior?
- What other information, or data, would you want to be readily available to you to monitor the patients’ learning of pressure injury prevention behaviors?
  For example, would it help you to know how many pressure reliefs the patient conducted during the previous hour?
- Would you like the data available for you to be in a particular format?
  For example, an average number of pressure reliefs conducted over the past four hours, or the percentage of offload over a certain period?
  How far back would you want data available for monitoring or educating pressure relieving behaviors?
  Do you want the information pushed to you?
- Can you think of any data that would not be helpful for you to be able to access?
  For example, when reviewing the patients pressure relieving behavior would you find it useful to know the actual peak psi’s that occurred over the previous period of monitoring, or would that be too much information?

### Implementation Strategies

We developed the draft of our study-specific implementation strategies (Table 1). From this chart, a refined, operational implementation guide would be created.

**Table 1:** Implementation strategies chart.

Study phase	Implementation strategies
Prestudy	Conduct local needs assessment Conduct local consensus discussions Fund and contract for the clinical innovation Use an implementation advisor Develop an implementation glossary Identify and prepare champions Identify early adopters Inform local opinion leaders Build a coalition Assess for readiness and identify barriers and facilitators Promote network weaving Develop and organize quality monitoring systems Develop and implement tools for quality monitoring
Aim 1: Clinical system development: define scope of implementation of AW-Shift^a^ into a Connected Care model	Develop a formal implementation blueprint Build a coalition Conduct educational meetings Distribute educational materials Promote adaptability Prepare patients or consumers to be active participants Involve patients or consumers and family members Obtain and use patients or consumers and family feedback Shadow other experts Use data warehousing techniques
Aim 2: Rapid cycle quality improvement: rapid quality improvement assessment of the implementation plan	Facilitation Conduct educational meetings Conduct cyclical small tests of change Provide ongoing consultation Provide local technical assistance Provide clinical supervision Organize clinician implementation team meetings Prepare patients or consumers to be active participants Involve patients or consumers and family members Purposely reexamine the implementation Obtain and use patients or consumers and family feedback Tailor strategies Remind clinicians Intervene with patients or consumers to enhance uptake and adherence Shadow other experts Facilitate relay of clinical data to providers Use data warehousing techniques
Aim 3: Hybrid pilot clinical and implementation trial: randomized controlled trial to test whether AW-Shift system adds value to clinical care of people with SCI/D^b^	Stage implementation scale up Provide ongoing consultation Provide local technical assistance Provide clinical supervision Remind clinicians Recruit, designate, and train for leadership Use data warehousing techniques Obtain formal commitments

^a^AW-Shift: Assisted Weight-Shift.

^b^SCI/D: spinal cord injuries and related disorders.

## Results

This study was funded in January 2023, and the IRB approval for Aim 1 of the study was December 5, 2022. We have recruited 6 participants into the clinician arm of our Aim 1 qualitative study as of manuscript submission. Expected results for Aim 1 to be published in winter 2024.

## Discussion

### Expected Outcome

Pressure mapping performed in rehabilitation clinics can effectively determine appropriate equipment and positioning in a wheelchair [42-44], but the translation of the same information from daily life into clinical care has not been previously tested. The day-to-day uncertainty of ulceration leads to patient appointments simply to “be mapped” as a preventative action often involving long distances and, sometimes, with an active pressure injury. A system such as AW-Shift can be integrated into a connected care model to allow for the integration of real-world data into the clinical practice of wheelchair users with SCI/D. The system has the potential to enhance the prevention of pressure injuries. Our mapping system has the ability to address and correct the contributors to pressure ulceration that have been observed in seating and mobility clinics: undetected cushion failures; caregivers who are unexpectedly late or absent, leaving the patient in the chair longer than necessary; frequent caregiver turnover; inexperienced caregivers; and caregivers who struggle with positioning the patient. AW-Shift can provide meaningful information about the daily life of people with SCI/D to the patient, caregivers, and clinicians to significantly improve the management of patient needs.

This project will demonstrate the ability to use an mHealth solution for improvement in pressure injury clinical outcomes. The success of this project will have sustained, powerful influence in the field of SCI/D through enhanced evidence of pressure injury risks that are present in a wheelchair user’s daily routine and environment that are not identifiable through routine, in-person evaluations in the clinic. It will further demonstrate the opportunity for the clinician and individual with SCI/D to work together to plan effective strategies for more expeditious interventions.

We hypothesize that an enhanced connected model of care using remote monitoring of pressure management will result in improved clinical outcomes (wound recurrence, early identification of wounds, and wound healing) for adults with SCI at high risk for pressure injury recurrence. We have performed extensive studies assessing the acceptability, usability, and efficacy of the AW-Shift system with adults with SCI/D. We now need to partner with patients and clinicians (therapists) to capture seating pressure information during daily life to inform clinical visits for individualized seating and skin health recommendations. On the basis of our projects funded by DoD, NIH, and Veterans Administration, we know that adults with SCI/D and therapists who treat them have the strong desire for a visual system and expanded access to data from daily life. Changing current clinical practices will require engagement and collaboration with providers and patients about what information is critical and how it should be used with clinical guidelines. This project will expand upon our previous study to move the AW-Shift system into routine clinical care, which was a high desire of adults with SCI/D for improved individualized care plans to prevent pressure injuries.

## Abbreviations

AW-Shift: Assisted Weight-Shift
CFIR: Consolidated Framework for Implementation Research
DoD: Department of Defense
IRB: institutional review board
mHealth: mobile health
NIH: National Institute of Health
PI: principal investigator
REDCap: Research Electronic Data Capture
SCI/D: spinal cord injuries and related disorders
SCI: spinal cord injury
